# Imaging of Bubonic Plague Dynamics by *In Vivo* Tracking of Bioluminescent *Yersinia pestis*

**DOI:** 10.1371/journal.pone.0034714

**Published:** 2012-04-05

**Authors:** Toan Nham, Sofia Filali, Camille Danne, Anne Derbise, Elisabeth Carniel

**Affiliations:** Yersinia Research Unit, Institut Pasteur, Paris, France; University of Louisville, United States of America

## Abstract

*Yersinia pestis* dissemination in a host is usually studied by enumerating bacteria in the tissues of animals sacrificed at different times. This laborious methodology gives only snapshots of the infection, as the infectious process is not synchronized. In this work we used *in vivo* bioluminescence imaging (BLI) to follow *Y. pestis* dissemination during bubonic plague. We first demonstrated that *Y. pestis* CO92 transformed with pGEN-*luxCDABE* stably emitted bioluminescence *in vitro* and *in vivo*, while retaining full virulence. The light produced from live animals allowed to delineate the infected organs and correlated with bacterial loads, thus validating the BLI tool. We then showed that the first step of the infectious process is a bacterial multiplication at the injection site (*linea alba*), followed by a colonization of the draining inguinal lymph node(s), and subsequently of the ipsilateral axillary lymph node through a direct connection between the two nodes. A mild bacteremia and an effective filtering of the blood stream by the liver and spleen probably accounted for the early bacterial blood clearance and the simultaneous development of bacterial foci within these organs. The saturation of the filtering capacity of the spleen and liver subsequently led to terminal septicemia. Our results also indicate that secondary lymphoid tissues are the main targets of *Y. pestis* multiplication and that colonization of other organs occurs essentially at the terminal phase of the disease. Finally, our analysis reveals that the high variability in the kinetics of infection is attributable to the time the bacteria remain confined at the injection site. However, once *Y. pestis* has reached the draining lymph nodes, the disease progresses extremely rapidly, leading to the invasion of the entire body within two days and to death of the animals. This highlights the extraordinary capacity of *Y. pestis* to annihilate the host innate immune response.

## Introduction

Plague is an infectious disease caused by the Gram-negative bacillus *Yersinia pestis* [1]. The incidence of the disease has gradually decreased during the course of the third pandemic that started from Hong Kong in 1894. However, since the 1990's, human plague reappeared in countries where no cases had been reported for decades, and thus the plague is now categorized as a re-emerging disease [2]. Rodents are the main plague reservoir and the disease is transmitted from rodent to rodent via the bite of infected fleas [3]. Humans, which are incidental hosts of *Y. pestis*, are most commonly infected after the inoculation of the bacillus into the dermis during a fleabite. This mode of *Y. pestis* penetration generally gives rise to the development of the most common form of infection, bubonic plague, which is characterized by the appearance of a painful and inflammatory lymph node, the pathognomonic bubo. The infection then spreads to various organs, leading to a terminal and fatal septicemia. In the absence of treatment, bubonic plague lethality varies from 50 to 70% [4].

Various animal models are utilized to study plague pathogenesis (mice, monkeys, rats, etc.) [5]–[9], the most widely used being the mouse experimental model. This model is appropriate and reliable to study a natural *Y. pestis* infection, as rodents are the normal hosts for *Y. pestis*. To follow disease progression, the conventional method is to experimentally infect mice with *Y. pestis*, sacrifice groups of animals at various time intervals, and determine bacterial counts in their tissues. This methodology has brought valuable information about the mode of *Y. pestis* spread during the infectious process, but it is laborious and time consuming, as it requires to collect various organs at various times post-infection, to crush them, to prepare serial dilutions of the tissue suspensions, to streak them on agar plates, and to count the bacterial colonies. Moreover, with this method, bacterial dissemination can be followed only in the tissues that have been collected. Most importantly, this approach suffers from a serious drawback attributable to intrinsic variations of biological systems. Indeed, the kinetics of bacterial spread and the fatal outcome greatly vary from one animal to another, even when inbred mouse strains are used. It is thus not possible to accurately follow disease progression as, at a given time of sacrifice, the animals are at different phases of the pathological process. One way to partly circumvent this biological variability is to use large cohorts of animals in order to obtain statistically significant data. This approach may bring reliable results, but is labor-intensive, expensive, and goes against the Animal Care standards and the current trend to reduce as much as possible the number of animals used for experimental studies. Other ways to limit biological variability include the injection of bacteria at very high doses or through artificial routes (intravenously or intraperitoneally), thus allowing a more rapid bacterial spread and therefore a narrower window of variation. However, these non-physiological doses or modes of penetration generate pathological processes that do not accurately reflect the normal course of the bubonic infection [10].

The recent advent of *in vivo* bioluminescence imaging (BLI), that allows tracking the spread of inert particles or microorganisms in live animals, has been a step forward in the study of physiological and pathological processes. This technology has been successfully applied to the analysis of various viral, bacterial and fungal infections [11]–[14], including diseases caused by category A biowarfare bacterial agents such as *Bacillus anthracis* [15] and *Francisella tularensis* [16]. This technique relies on the introduction into a bacterium of genes encoding an enzyme that, in the presence of its substrate, catalyzes a reaction generating bioluminescence. The light emitted by the recombinant microorganism is measured using an *in vivo* BLI system. The bioluminescence tool has recently been applied to members of the genus *Yersinia*. For instance, the impact of a partial deletion of the pYV-encoded YopE protein on the course of a *Yersinia pseudotuberculosis* infection in BALB/c mice was followed using recombinant bacteria carrying on the pYV plasmid, the *luxCDABE* locus that encodes the luciferase and its substrate [17]. More recently, monitoring of gene expression during a *Y. pseudotuberculosis* infection was made possible after inserting various gene promoters upstream of the *luxCDABE* operon [18]. In *Yersinia enterocolitica*, a strain in which the *luxCDABE* operon has been inserted into the chromosome allowed tracking of the bacteria in living animals and comparison of the fate of the wild type strain to that of an *inv* mutant derivative [19].

In this study we first determined whether a bioluminescent *Y. pestis* could be a reliable tool to perform *in vivo* imaging, and we subsequently used this recombinant strain to study bubonic plague progression in a mouse model of experimental infection.

## Results

### Construction of a bioluminescent *Y. pestis* strain

**Figure 1:**
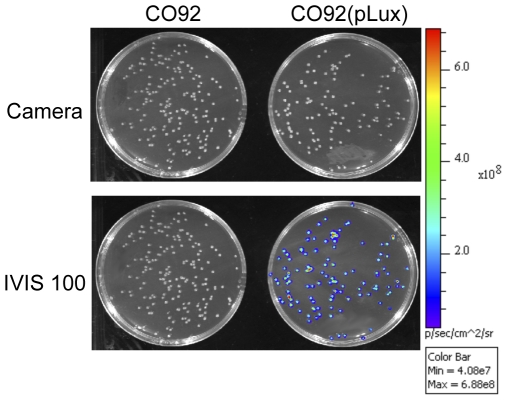
Bioluminescence imaging of *Y. pestis*. *Y. pestis* CO92 or CO92(pLux) were grown for 48 h at 28°C on LB-Carb plates. The plates were placed in an IVIS 100 device dark chamber, photographed under normal light and then bioluminescence emission was detected using a 7 s exposure time and a picture binning of 4. Both pictures were superimposed by the Living image software. Colored dots represent bioluminescence emitting bacteria. The color bar on the right shows the intensity of bioluminescence light coded in the picture from indigo (4.1×10^7^ photon/s.cm^2^.steradian) to red (6.9×10^8^ photon/s.cm^2^.steradian).

A bioluminescent clone of *Y. pestis* was obtained by introduction of plasmid pGEN-*luxCDABE*, which harbors the *luxCDABE* operon from *Photorhabdus luminescens* under the control of the P*em7* promoter [12] into the virulent *Y. pestis* strain CO92, yielding CO92(pLux). Bioluminescence emission by the recombinant strain grown on LBH-Carb agar plates was checked with the IVIS 100 device. A luminescence signal was emitted by all CO92(pLux) colonies on the plate, while no signal was detected in the CO92 wild-type strain (Figure 1). Similarly, CO92(pLux) but not CO92 grown in broth emitted a bioluminescence signal detected with a Xenius plate reader (data not shown). The *luxCDABE* operon is thus functional in *Y. pestis*.

### Evaluation of the bioluminescence tool under *in vitro* conditions

Since exogenous plasmids may impair *Y. pestis* multiplication, the growth kinetics in LB broth of CO92 and CO92(pLux) were followed over a two-day period. Both strains exhibited similar growth kinetics (Figure S1). A slight delay in bacterial multiplication was observed between 24 h and 28 h of growth. However, this growth delay disappeared after 30 h (P≥0.2 for the median cfu of CO92 and CO92(pLux) according to the Mann Whitney test).

The linear relation between light intensity and cfu counts was calculated using a large range of bacterial concentrations. For this purpose, CO92(pLux) was grown on LBH-Carb agar plates at 28°C, suspended in LB broth, and serially diluted from 7.5×10^1^ to 7.5×10^8^ cfu/ml. Each dilution was used to quantify the intensity of bioluminescence produced. As shown on Figure S2.A, a linear relationship between cfu and light emission was obtained for bacterial concentrations ≥7.5×10^4^ cfu/ml. After logarithmic transformation of light intensity and cfu counts ≥7.5×10^4^ cfu/ml, the Pearson two-tailed test indicated a highly significant correlation (P<0.0001). Light signals generated by bacterial concentrations below 7.5×10^4^ cfu/ml represented the background level, which was around 175 photons/s.ml. The correlation between cfu and light emission was further analyzed in actively multiplying bacteria. Aliquots of CO92(pLux) growing in LB-Carb broth were taken at various time points to both quantify bioluminescence emission and determine cfu counts. A robust correlation (P<0.0001) between the two sets of data was again observed (Figure S2.B). The intensity of the bioluminescent signal could thus provide an accurate estimate of the number of cells for bacterial concentrations ≥7.5×10^4^ cfu/ml.

The *luxCDABE* operon is under the control of the P*em7* promoter, which is constitutive in *E. coli* [12]. Since *Y. pestis* has to adapt to different environments and temperatures during its life stage in the flea (21–30°C) and in a mammalian host (37°C), we considered whether expression of *luxCDABE* would be constitutive in this species, or whether it would be temperature-dependent. As shown on Figure S3, the amount of light per cfu was significantly higher when bacteria were grown at 37°C than at 28°C, both on agar plates and in broth. These results suggest that the temperature of the mammalian host might be favorable for an efficient production of luciferase activity.

The stability of pGEN-*luxCDABE* and of the bioluminescence signal over time and bacterial division was determined by performing 13 subcultures of CO92(pLux) in LB broth without carbenicillin over 19 days. At different time points (day 0, 3, 5, 7, 10, 12, 14, 17 and 19), bacterial aliquots were streaked on LBH plates and individual colonies were checked for their capacity to grow in the presence of Carb (maintenance of the pGEN-*luxCDABE* plasmid), and to emit luminescence (maintenance of the luciferase activity). The proportion of pGEN-*luxCDABE*-harboring colonies remained high (>95%) throughout the experiment, and all emitted light (data not shown), indicating a high stability of bioluminescence emission in CO92(pLux) under *in vitro* conditions.

### Evaluation of the bioluminescence tool under *in vivo* conditions

The potential impact of the presence of pGEN-*luxCDABE* on *Y. pestis* virulence was evaluated in the mouse model of bubonic plague. The presence in the recombinant CO92(pLux) strain of the major virulence factors that are easily lost in *Y. pestis* (pPla, pYV and the HPI) was confirmed by PCR prior to infections. The LD_50_ by the subcutaneous (sc) route of CO92(pLux) was similar to that of the wild-type CO92 (<10 cfu for both strains). The survival curves were also similar (Figure S4), with no statistical difference (Mantel-Cox test) in animals infected with 10 cfu (P = 0.63) or 100 cfu (P = 0.19) of either strain, indicating that the presence of pGEN-*luxCDABE* did not affect the virulence of *Y. pestis* upon sc infection.

The maintenance of the plasmid and of the capacity of CO92(pLux) to emit light upon *in vivo* multiplication was verified by analyzing bacteria recovered from the spleen and liver of two moribund mice on day 5. All 300 colonies tested (200 recovered from the spleen and 100 from the liver) grew on LBH-Carb plates and emitted light (data not shown), indicating that the plasmid and the luminescence activity were retained during *in vivo* growth of *Y. pestis*.

**Figure 2:**
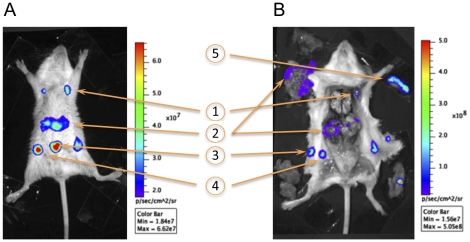
Linking a bioluminescence signal to the colonization of a specific organ. (A) Example of the light emitted by a live animal. (B) Light emitted by the organs of the same animal. Arrows point at: (1) axillary lymph nodes, (2) liver (one lobe still in place and the remaining of the organ removed from its anatomical site), (3) injection site (split in two parts during dissection), (4) inguinal lymph nodes, and (5) spleen (removed from its anatomical site and visualized in live animals mostly in ventral position). On the dissection picture, the gut has been removed and placed next to the right hind leg, while the peritoneum including a portion of the infection site is located next to the left hind leg. Color bars on the right of each picture show the intensity of bioluminescence light coded in the picture from indigo to red.

To determine whether the bioluminescence signal emitted by *Y. pestis* was sufficient to be detected during the infectious process, mice were infected sc with 100 cfu of strain CO92(pLux). The animals were anesthetized and monitored daily for bioluminescence emission with the IVIS 100 device. A signal was visible at the injection site from the first observation (24 h post-infection) and appeared in other locations in the following days (Figure 2A), indicating that the bioluminescent signal emitted by CO92(pLux) could be used to track *Y. pestis* dissemination *in vivo*.

To link the signal sources to the colonization of specific organs, infected mice were bioluminescence-monitored at different times post-infection (pi). They were subsequently sacrificed, dissected to expose the organs, and monitored again. The luminescent signals observed in live animals in a dorsal position matched those of the anatomical sites (injection site, liver, axillary and inguinal lymph nodes) from which the signal was predicted to be emitted (Fig. 2B). Bacterial colonization of the spleen was best visualized when the animals were placed in ventral position.

**Figure 3:**
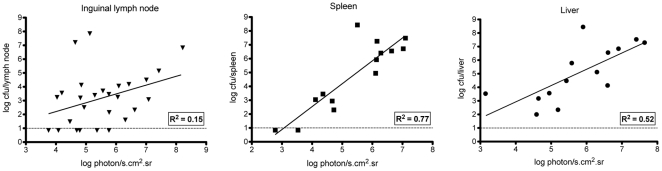
Correlation between bioluminescence emission by specific organs in live animals and cfu counts. Live animals were monitored for bioluminescence emission and subsequently sacrificed at various times pi. Their inguinal lymph nodes, spleen and liver were taken to determine the bacterial load in each organ. Each point accounts for one organ taken from an infected animal. The continuous line represents the least square fit linear regression, and the related goodness of fit coefficient (R^2^) is indicated on each graph. The horizontal dotted line indicates the detection limit of the bacterial load (10 cfu).

Next we wanted to determine whether the amount of bioluminescence emitted from the inguinal lymph nodes, spleen and liver of live animals correlate with bacterial loads. For this purpose 14 mice were infected sc with 100 cfu of CO92(pLux), they were bioluminescence-monitored at different time points, and the radiance from the region of interest (ROI) defining each organ was measured. The animals were immediately sacrificed afterwards and their organs were removed and crushed to determine cfu counts. Light emission increased with bacterial burden in the three organs (Figure 3), and according to the Pearson test, the number of bacteria significantly correlated with the intensity of light emission in these tissues (inguinal lymph nodes: P = 0.0392, spleen: P<0.0001, liver: P = 0.0017). The higher P value observed for the inguinal lymph nodes might be at least partly attributable to several nodes that emitted light while no cfu were obtained (triangles below the detection limit on Figure 3A). This could be due to a technical problem linked to the difficulty to differentiate the inguinal lymph nodes from the surrounding fat tissue in some mice. A removal of these probably falsely cfu-negative nodes would have further improved the correlation between bacterial loads and light emission. Nevertheless, our results indicate that bioluminescence quantification can provide reliable non-invasive measures of *Y. pestis* burden in the organs of infected live animals.

To obtain data that could be used to compare disease progression between mice, as well as at different times pi in the same mouse, a predefined standard setting ranging from 7×10^5^ to 5×10^8^ p/sec/cm^2^/sr was selected and used for all *in vivo* measurements of bioluminescent signals in live animals. This range of radiance was chosen to avoid most of background light (lower setting high enough), while reflecting a wide dynamic scale (the highest upper setting that still permitted contrast). Using this predefined setting, the minimal amounts of bacteria that allowed a signal detection were 8.3×10^5^ cfu for the spleen, 1.3×10^5^ cfu for the liver, and 1.5×10^4^ cfu for the inguinal lymph nodes.

### General scheme of *Y. pestis* dissemination *in vivo*

Once the characteristics of the bioluminescent tool were defined and its suitability to track *Y. pestis* in live animals confirmed, we used this tool to follow the course of bubonic plague in individual animals. BALB/cByJ female mice were infected sc in the *linea alba* with 100 cfu of CO92(pLux) on day 0 (D0), and the animals were anesthetized and monitored daily for bioluminescence emission from D1 until their death, using our defined IVIS settings. To limit the effect of individual variations inherent to live biological systems, a large group of mice (46 animals) was used in six independent experiments, and the data were pooled and analyzed.

**Figure 4:**
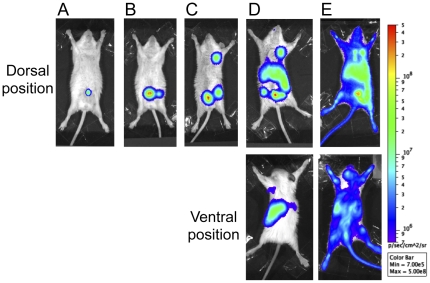
Different steps of bacterial spread during bubonic plague. The bioluminescent signal was visible first at the injection site (A). It then reached the inguinal lymph node (B), the axillary lymph node (C), the liver (D, dorsal position) and the spleen (D, ventral position), and finally the entire body (E). Each picture is an example of an animal displaying a signal characteristic of each step. The color scale on the right represents the settings used to monitor light emission in all mice throughout the observation period.

**Figure 5:**
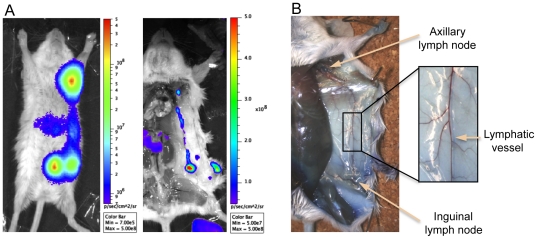
Lymphatic connection between the inguinal and axillary lymph nodes. (A) Observation of a bioluminescent signal connecting the two lymph nodes on a live and on a necropsied mouse. (B) Staining of the lymphatic system after injection in the *linea alba* of the Evans blue dye. The axillary and inguinal lymph nodes are stained in blue and the lymphatic vessel draining the two nodes is seen as a thin pale blue line adjacent to the blood vessels.

The bioluminescent signal followed the same route of spread in most animals (74%), allowing to delineate successive steps and to draw a general scheme of dissemination. An example of each of these steps is shown on Figure 4. A signal, already visible in all animals on the first day of observation (D1), was initially limited to the site of injection (Figure 4A). The second site to emit light was the inguinal lymph node either on one side or on both sides of the animal (Figure 4B). The following luminescent organs were the ipsilateral axillary lymph nodes (Fig. 4C). A light-emitting line running between the inguinal and axillary lymph nodes was sometimes visible both on live and necropsied animals (Figure 5A), suggesting a direct connection between the two lymph nodes via the lymphatic vessels. To visualize this route of lymphatic drainage, 100 µl of a 5% Evans Blue solution were injected sc in the *linea alba* of four mice. The animals were sacrificed 1, 10, 20 or 30 min after the injection to follow the fate of the dye. The two lymph nodes were stained in blue and a thin, pale vessel also colored in blue and connecting the two lymph nodes was visible adjacent to the blood vessels (Fig. 5B). These results are consistent with a route of bacterial dissemination starting from the injection site and using the lymphatic stream to reach first the inguinal lymph node and then the axillary lymph node. Next, the bioluminescence extended to the sub diaphragmatic zone identified as the liver region, and a spot corresponding to the spleen was also visible on the back of the animals (Figure 4D, dorsal and ventral position). A bioluminescence of the entire animal, including peripheral sites such as the tail or the ears (Figure 4E), highly evocative of a septicemic stage was then observed. This terminal septicemia, which was confirmed by the recovery of circulating *Y. pestis* from the blood, was systematically followed by the death of the animals.

Exceptions to this general scheme were a limitation of the signal to the injection site until the animal death (11% of the mice) or a direct colonization of the inner organs without visualizing the lymph node steps in 15% of the animals, both suggestive of a direct passage of the bacteria in the blood.

Of note, no signal at anatomical sites corresponding to the intestine, the lungs or the kidneys were detected on living animal before the final septicemic phase. This was in accordance with the absence of bioluminescence detected in these organs when animals were autopsied (Figure 2B).

### Kinetics of bacterial progression during bubonic plague

**Figure 6:**
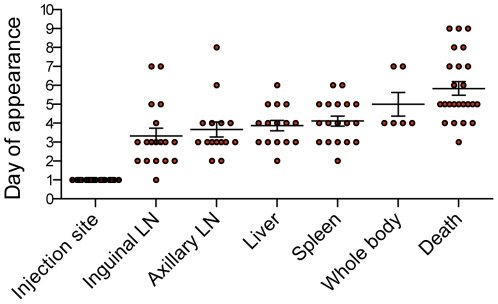
Time of appearance of a bioluminescent signal in the various organs of infected mice. Each circle corresponds to an individual animal. Horizontal bars represent the mean and vertical bars the standard error of the mean of the data collected from 24 mice. LN: lymph node.

To determine the kinetics of bacterial spread in our mouse model of bubonic plague, the average time at which the anatomical sites started to emit light was calculated for 24 individual mice for which all data were available from D1 until their death. The injection site was always bioluminescent from the first day of observation (D1) in all animals. On average, the signal appeared on D3 in the inguinal lymph node (D3.3±0.4) and soon after in the axillary lymph node (D3.6±0.4) (Figure 6). The next affected organs were the spleen and liver, which both started to emit light around D4 (4.1±0.3 and 3.9±0.3, respectively). Their colonization was followed on D5 (5±0.6) by a bioluminescence of the entire body, characteristic of a septicemic phase. Because of the very short period of time elapsed between invasion of the whole body and death, the septicemic phase could not always be observed with our daily monitoring window. The death of the animals occurred on average around D6 (5.8±0.4).

This chronology of bacterial dissemination allowed estimating of the average time at which the colonization of each organ occurred in a group of animals. However, our observations also evidenced important differences from one animal to another. The constant feature was that all mice emitted light at the site of injection on D1, and that the signal was restricted to this site in all except one animal at this early time point (Figure 6). After D1, large variations in the times at which the various colonization steps occurred were observed. For instance, the time of the first light emission in the inguinal lymph node varied from D1 to D7 in different animals. Similarly, a variation of 6 days was observed for the appearance of bioluminescence in the axillary lymph node, of 4 days in the spleen and liver, of 3 days for the whole body and of 6 days for the fatal outcome. These results reflect a high variability in the rapidity of dissemination of the bacteria in each animal.

Despite this variability, the analysis of the time interval elapsed between the appearance of a signal at two consecutive steps during disease progression for each individual mouse revealed a remarkably consistent trend. Although emission of light from the inguinal lymph nodes could take up to 7 days, once this site started to emit light, the kinetics of dissemination to other organs was remarkably constant in all animals. Indeed, from the lymph nodes, the signal reached the liver and spleen within one day (1±0.2), the entire body (septicemic phase) in two days (2±0.5), and the animal died soon after (D2.2±0.3). All animals seen at the septicemic phase were systematically found dead on the next day. In a few cases, the disease progressed even faster and the window frame of one day did not allow to visualize the intermediate steps between lymph node colonization and the terminal phase of the infection. Therefore, our results revealed two important phenomena: (i) the variations in the kinetics of bacterial spread were essentially attributable to the length of time the signal remained limited to the injection site, and (ii) as soon as the signal reached the lymph nodes, the disease progressed very rapidly, leading to the animal death within two days.

## Discussion

Past studies performed at the post-mortem stage on autopsied human plague victims have been invaluable for understanding pathological processes that took place during bubonic plague. However, they were performed after the terminal phase of the disease, when the entire body was colonized, and therefore the mode of *Y. pestis* dissemination could not be clearly established. Works using various animal models brought additional and useful information. However, they only give snapshots of the infection, as the infectious process is not synchronized in all animals. Indeed, follow up of bacterial infection progression is most often hampered by the biological variability inherent to animal models. This phenomenon is exemplified by the fact that the LD_50_, commonly used as a means for quantifying bacterial virulence, relies on the fact that a similar bacterial inoculum will be lethal for only half of the animals, even if they have an identical genetic background (syngenic mice) and are kept under similar conditions. In addition, when physiological doses and routes of inoculation are utilized, variations in time to death are commonly observed. This lack of synchronization makes the analysis of bacterial spread, tissue lesions or innate immune response difficult, as the animals are not at the same phase of the infectious process at the time of sacrifice. Tracing bacterial spread through the monitoring of a bioluminescent signal can bring a technical answer to this issue. The use of *in vivo* imaging has been applied to the study of various bacterial infections and has revealed some pathophysiological processes that had not been previously seen using conventional methods [12], [15]. This technique is much simpler and less time consuming than methods relying on bacterial isolation and enumeration from different organs. It has also the advantage over conventional methods based on the sacrifice of groups of animals at given time points to make possible both reduction and refining of animal use, two of the ‘3R principles’ of Russell and Burch [20].

The aim of this work was to use the *in vivo* BLI technology to gain a deeper knowledge of the mode of *Y. pestis* dissemination during bubonic plague. Since bioluminescence has never been used to track *Y. pestis* during the infectious process, the first step of this study was to determine its applicability to the plague bacillus. The capacity of *Y. pestis* to emit bioluminescence *in vitro* was previously shown after insertion of the *luxAB* genes downstream of the *ymt* or *caf* promoters in strain EV76 [21]. Similarly, we observed here that a *Y. pestis* CO92 carrying pGEN-*luxCDABE*, a low-copy number plasmid that encodes the luciferase enzyme and its substrate [12], gained the capacity to emit light during growth in broth or on agar plates.

However, this technology would only be applicable if the plasmid carrying the *lux* operon is stable and if the bioluminescent signal does not wane with bacterial division *in vitro* or *in vivo*. We found that the pGEN-*luxCDABE* plasmid was kept and the luciferase activity was retained in 95% of the colonies despite repeated *Y. pestis* subcultures *in vitro*, and in 100% of the colonies upon multiplication in mouse organs. Introduction of exogenous plasmids into *Y. pestis* may impair its virulence [22]. Moreover, the product of *luxD* has been found to be toxic in mycobacteria, most likely because, in conjunction with the products of *luxC* and *luxE*, it forms part of an enzyme complex which alters the cell wall structure of these bacteria [23]. We found that neither the presence of the pGEN-*luxCDABE* plasmid, nor the luciferase activity had an impact on the kinetics of bacterial growth *in vitro* and on the virulence of *Y. pestis* in the mouse experimental model of bubonic plague.

The luciferase activity produced by *Y. pestis* CO92(pLux) during the infectious process was high enough to generate a light signal that was detectable by the IVIS. Moreover, the assignment of a specific organ to each light spot based on its anatomical location in live mice was confirmed after animal dissection and organ exposure, thus indicating that the bioluminescent signal could be used to track *Y. pestis* progression in the mouse experimental model of bubonic plague. In other infection models, the intensity of light emission did not systematically give an accurate estimate of bacterial loads in various organs. While a good correlation between these two parameters was observed during inhalational and intestinal anthrax [15], or in the neutropenic mouse thigh model of *Escherichia coli* infection [24], this was not the case in the *Streptococcus pneumoniae* lung infection model for instance [25]. We found here that *Y. pestis* cfu counts and light intensity emitted by infected organs (lymph nodes, spleen and liver) in live mice were significantly correlated, thus providing a reliable picture of the route of bacterial dissemination.

To study the kinetics of bacterial spread in the same animal and to compare disease progression in different animals and at different time points, we decided to use a predefined standard light setting that was not too sensitive to minimize background luminescence while providing a good contrast between light emitting spots. Using this setting, the detection limit ranged from ≈10^4^ to 8×10^5^ cfu depending on the organ. Hence, an absence of light emission in an organ means that the bacterial load is below this threshold, and not necessarily that there is a complete absence of bacteria in this organ. Similar thresholds have been reported in the rat endocarditis model of *Staphylococcus aureus* infection [26].

Having demonstrated that a bioluminescent *Y. pestis* strain could be used for *in vivo* BLI, we applied this technology to the analysis of bubonic plague development in live mice. The use of a large set of animals allowed us to confidently draw a general scheme of *Y. pestis* dissemination. Our results indicate that the first step of the infectious process is a bacterial multiplication at the site of injection in the *linea alba*. This step is followed by a colonization of the draining inguinal lymph node, in accordance with the clinical descriptions of bubonic plague in humans, characterized by a bubo draining the site of the flea bite [4]. Since we performed the sc injection approximately in a median line on the abdomen, either a unilateral or a bilateral infection of the inguinal lymph nodes was observed. The next site colonized is the ipsilateral axillary lymph node. This route of dissemination from the initial draining lymph node (designated primary bubo of first order) to the next one located on the same side (primary bubo of second order) was suggested by pathological examinations of patients who died of bubonic plague [4], [27]. Since these analyses were done at a post-mortem stage characterized by an invasion of the whole body, the direct migration from the initial bubo to the contiguous lymph node could not be fully established. The fact that we observed a bioluminescent line connecting the two lymph nodes at an early stage of the infection demonstrates that this route of spread is correct. The existence of known lymphatic connections between the two nodes [28], and our visualization of lymph vessels linking them, strongly suggest that the bacteria use the lymphatic stream to migrate from one lymph node to the next one. This route is also supported by the observation that in a rat model of bubonic plague, the bacteria penetrate into the primary bubo of second order (corresponding to the ipsilateral axillary lymph node), through the marginal sinus, which is drained by the afferent lymph channels [9].

Next the infection progresses to the liver and spleen, which are colonized almost simultaneously. This is consistent with previous works reporting high bacterial loads in these organs in human plague victims and animal models [4], [9], [27], [29]. The colonization of these lymphoid tissues requires a passage of the bacteria into the blood circulation, either after a bacterial discharge into the thoracic duct, or directly within the infected lymph nodes by *in situ* disruption of the blood vessels. Arguing for the latter mode of blood invasion is the observation of major hemorrhages with disrupted blood vessels in the primary lymph nodes of infected mice or rats [6], [9]. This early bacteremia should be moderate and transient, as no diffuse bioluminescent signal characteristic of a heavy blood infection was observed prior to the infection of the spleen and liver. An effective filtering of the blood stream by these lymphoid organs may explain the early clearance of the bacteria from the blood circulation and the ensuing development of secondary bacterial foci within these organs. The saturation of the filtering capacity of the spleen and liver coupled with massive *in situ* bacterial multiplication subsequently leads to a terminal septicemia with an invasion of the entire body, as attested by a luminescent signal emitted from the whole animal. This terminal septicemic stage of bubonic plague has been largely documented, both in human victims [4], [27], [30], [31] and in non-human hosts [5], [8], [9], [29], [32], [33].

It is worth noting that anatomical sites other than secondary lymphoid tissues did not emit light until the terminal septicemic phase in live animals. Previous studies that quantified bacterial loads in various tissues of experimentally infected animals showed for instance that the lungs could be infected. When we performed cfu counts from the lungs of necropsied animals, we could also detect the presence of bacteria, but most of the time they were present in low amounts (<10^4^ cfu), and therefore below the detection limit. Therefore, although this non-lymphoid organ could be colonized, it does not seem to be a major site of *Y. pestis* multiplication. It is possible that the bacteria recovered from the lungs were from animals at the beginning of the systemic phase of the infection. Altogether, *in vivo* BLI suggests that the main targets of *Y. pestis* multiplication are the secondary lymphoid tissues (lymph nodes, spleen and liver), and that colonization of other organs is much less pronounced and may result from a secondary septicemic spread, at the terminal stage of the infectious process.

The ability to follow the progression of the disease over time in the same animal also allowed us to better define the kinetics of infection. Our observations evidenced important animal-to-animal variations in the rapidity of bacterial dissemination. Remarkably, our analysis showed that the high individual variability in the chronology of disease progression was directly linked to the time the bacteria remained confined at the site of injection (1 to 6 days). Impressively, once the bacteria had reached the lymph nodes, the disease progressed extremely rapidly, leading to the invasion of the entire body (septicemic phase) within two days and soon after to the death of all animals. This highlights the extreme acuteness of bubonic plague and the extraordinary capacity of *Y. pestis* to disseminate through secondary lymphoid organs to cause an extremely rapid and fatal septicemia.

In conclusion, the *in vivo* imaging, by tracking the spread of *Y. pestis* in live animals, allowed us to draw a general scheme that delineates the successive steps and kinetics of bacterial dissemination. This tool may have other promising applications, such as the analysis of *Y. pestis* gene expression during disease progression, or the study of the impact of gene mutations on the course of the infectious process.

## Materials and Methods

### Bacterial strains and growth conditions

The fully virulent *Y. pestis* strain CO92 [34] and its derivatives were grown in Luria Bertani (LB) broth for 24 h, or on LB agar plates supplemented with 0.2% hemin (LBH) for 48 h at 28°C. When necessary, carbenicillin (Carb; 100 µg/ml) was added to the media. All *in vitro* experiments with *Y. pestis* were performed in a Biosafety Level 3 laboratory. *E. coli* strain CFT073(pGEN-*luxCDABE*) [12] was grown for 24 h at 37°C in LB-Carb broth.

### Construction of a bioluminescent *Y. pestis*

Plasmid pGEN-*luxCDABE* [12], which carries the *luxCDABE* operon and confers resistance to carbenicillin, was extracted from strain CFT073(pGEN-*luxCDABE*) and introduced by electroporation [35] into *Y. pestis* CO92, yielding CO92(pLux). Presence of pGEN-*luxCDABE* in Carb^R^ colonies was checked by PCR with primers 5′-CCAGGTTGAAATCTTTCCCG-3′ and 5′-CTTTTTGAACTAAAGAATAGGC-3′, which amplify a portion of the *luxC* gene. The maintenance of major and unstable virulence factors was also checked by PCR with primers amplifying a portion of Pla (5′-ATCTTACTTTCCGTGAGAAG-3′; 5′-CTTGGATGTTGAGCTTCCTA-3′), the pYV (5′-ATAACTCATCGGGGGCAAAAT-3′; 5′-GCGTTATTTATCCGAATTTAGC-3′ that target *yopM*), or the High Pathogenicity Island (HPI) (5′-ATGCTGCATATCGCCTTTCGCCCCGAC-3′; 5′-GGACGTCGTGAATTTCGCAGGCGTTAGA-3′ that amplify *irp2*). Template DNA was obtained by suspending one bacterial colony into 100 µl of 50 mM sodium hydroxide and 0.25% sodium dodecyl sulfate, and heating at 95°C for 5 min. Each PCR reaction was done in a volume of 50 µl containing 2 µl of template DNA, 0.3 µM of each primer, 200 µM dNTP, 2 mM MgCl_2_, 1.25 U Taq Polymerase (Applied Biosystems) and 5 µl of 10× buffer. The PCR reactions comprised an initiation step of 5 min at 94°C; 35 cycles of 30 s at 94°C, 30 s at 55°C and 90 s at 72°C; and a termination step of 10 min at 72°C. Bioluminescence emission was detected and quantified with an In Vivo Imaging System (IVIS 100, Caliper Life Sciences) and a Xenius plate reader (SAFAS Monaco).

### Mouse infections

Animals were housed at the Institut Pasteur animal facility, which has been accredited by the French Ministry of Agriculture to perform experiments on live mice (accreditation B 75 15-01, May 22, 2008), in compliance with the French and European regulations on care and protection of Laboratory Animals (EC Directive 86/609, French Law 2001-486, June 6, 2001). The protocol used in this study (#03.99, May 13, 2009) was approved by the Hygiene & Security, and Veterinary Committees of the Institut Pasteur, and was performed in compliance with the NIH Animal Welfare Insurance (#A5476-01 issued on 02/07/2007).

Six- to eight-week-old BALB/cByJ mice (Charles River Laboratories) were maintained under specific pathogen-free conditions in a Biosafety Level 3 animal facility at the Institut Pasteur, in compliance with the European animal welfare regulations. Subcutaneous infections were performed by injecting 100 µl of bacterial suspensions in saline into the abdominal *linea alb*a. Bacterial counts were estimated by measuring the optical density at 600 nm and confirmed by plating the suspensions on LBH or LBH-Carb agar plates. The 50% lethal doses (LD_50_) were determined using groups of five mice infected sc with 10-fold serial dilutions of the bacterial suspensions. Animal deaths were followed for three weeks and the LD_50_ was calculated according to the method of Reed and Muench [36].

To quantify *Y. pestis* numbers in organs of infected animals or to study pGEN-*luxCDABE* and bioluminescence maintenance after *Y. pestis* multiplication *in vivo*, mice were infected sc with 100 cfu. At various time points pi, the animals were sacrificed, their organs were removed aseptically and crushed in a Mixer Mill MM 301 (Retsch). The suspensions were diluted in PBS and various dilutions were streaked on LBH plates. Individual colonies were streaked on LBH-Carb and light emission was determined with the IVIS 100 device.

### *In vivo* imaging

For *in vivo* tracking of *Y. pestis*, mice were infected sc with 100 cfu of CO92(pLux). They were anesthetized every day by intraperitoneal injection of 200 µl of a solution containing 20 µl of ketamine (Imalgene 1000) and 10 µl of xylazine (Rompun 2%) in saline. Mice were then set in a poly(methyl methacrylate) confinement box (TEM SEGA) and introduced in an IVIS 100 system (Caliper Life Sciences). Image acquisition and analysis were performed with the Living Image 3.2 software (Caliper Life Sciences), using acquisition times of 1 s to 240 s to allow maximal acquisition while avoiding signal saturation, and a binning set on ‘small’. To quantify the amount of light emitted by bioluminescent bacteria in a specific organ, the region corresponding to this organ (ROI or “region of interest”) was defined in the software and the average radiance of the defined region was calculated. To analyze and compare the signals in a large number of animals, a predefined setting ranging from 7×10^5^ p/sec/cm^2^/sr to 5×10^8^ p/sec/cm^2^/sr was used and applied to all measurements.

### Visualization of lymphatic drainage

To visualize the lymph nodes and the draining lymphatic vessels, a 5% (w/v) solution of Evans blue dye was injected into the mouse *linea alba*. After 1 to 30 min, the mice were sacrificed and the internal side of the skin extending from the lower to the upper limbs was exposed to visualize the axillary and inguinal lymph nodes.

### Statistical analyses

Statistical analyses and the plotting of graphs were performed using the software GraphPad Prism version 5.0d (San Diego, USA). For correlations, data of cfu counts and light radiances were processed as follows: after logarithmic transformation of both sets of values, normality of the sampling was controlled by a d'Agostino and Pearson omnibus normality test, and when the sample distribution was normal, a Pearson two-tailed correlation test was performed. When the correlation test was positive, a linear regression was performed using the least square fit method. Differences between bacterial concentrations or between ratios of photons/cfu in *in vitro* conditions were analyzed with the Mann Whitney test. Differences between survival curves were tested with a log-rank (Mantel-Cox) test.

## Supporting Information

Figure S1 **Comparison of the growth kinetics of CO92 and CO92(pLux).** CO92 (white circles) and CO92(pLux) (black squares) were grown for 48 h at 28°C in LB broth and aliquots were taken at various time points for bacterial counts. Shown are mean values and standard error of the means (vertical bars) of triplicate measures.
(PDF)

Figure S2 **Correlation between bioluminescence emission and cfu counts.** Bacteria were grown at 28°C on agar plates (A) before being suspended in LB and serially diluted, or in broth (B) and aliquots were taken at different time points to determine bacterial counts and light emission. The line represents the least square fit linear regression. The related goodness of fit coefficient (R^2^) is indicated on each graph.
(PDF)

Figure S3 **Temperature-dependent expression of** ***luxCDABE*** **in** ***Y. pestis*** **.** Bacteria were grown at 28°C (red circles) or 37°C (blue triangles), in LB broth or on LBH agar plates. Each spot represents the number of photons (count/s) emitted per cfu. The horizontal bar represents the mean light emission per cfu and the vertical bar the standard error. P values were determined with the Mann Whitney test.
(PDF)

Figure S4 **Survival curves of mice infected with** ***Y. pestis*** **CO92 or CO92(pLux).** Groups of five mice were infected subcutaneously with approximately 10 cfu of *Y. pestis* CO92 (red line) or bioluminescent *Y. pestis* CO92(pLux) (blue line) and their mortality was followed daily.
(PDF)
