# Supplementary material for: Imaging of Bubonic Plague Dynamics by In Vivo Tracking of Bioluminescent Yersinia pestis
Source: PLoS One. 2012 Apr 5;7(4):e34714. doi: 10.1371/journal.pone.0034714 (PMC3320629; doi:10.1371/journal.pone.0034714)
Supplement: Figure S1 — Comparison of the growth kinetics of CO92 and CO92(pLux). CO92 (white circles) and CO92(pLux) (black squares) were grown for 48 h at 28°C in LB broth and aliquots were taken at various time points for bacterial counts. Shown are mean values and standard error of the means (vertical bars) of triplicate measures. (PDF) [file pone.0034714.s001.pdf]

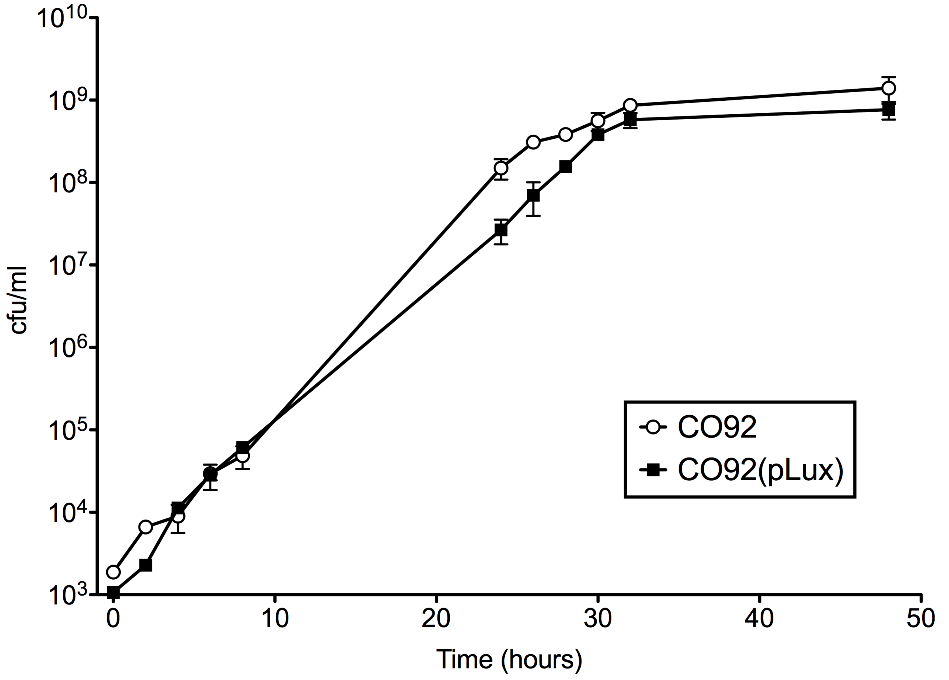

Figure S1. Comparison of the growth kinetics of CO92 and CO92(pLux).
CO92 (white circles) and CO92(pLux) (black squares) were grown for 48h at 28°C in LB broth and aliquots were taken at various time points for bacterial counts. Shown are mean values and standard error of the means (vertical bars) of triplicate measures.
