# Supplementary material for: Imaging of Bubonic Plague Dynamics by In Vivo Tracking of Bioluminescent Yersinia pestis
Source: PLoS One. 2012 Apr 5;7(4):e34714. doi: 10.1371/journal.pone.0034714 (PMC3320629; doi:10.1371/journal.pone.0034714)
Supplement: Figure S2 — Correlation between bioluminescence emission and cfu counts. Bacteria were grown at 28°C on agar plates (A) before being suspended in LB and serially diluted, or in broth (B) and aliquots were taken at different time points to determine bacterial counts and light emission. The line represents the least square fit linear regression. The related goodness of fit coefficient (R2) is indicated on each graph. (PDF) [file pone.0034714.s002.pdf]

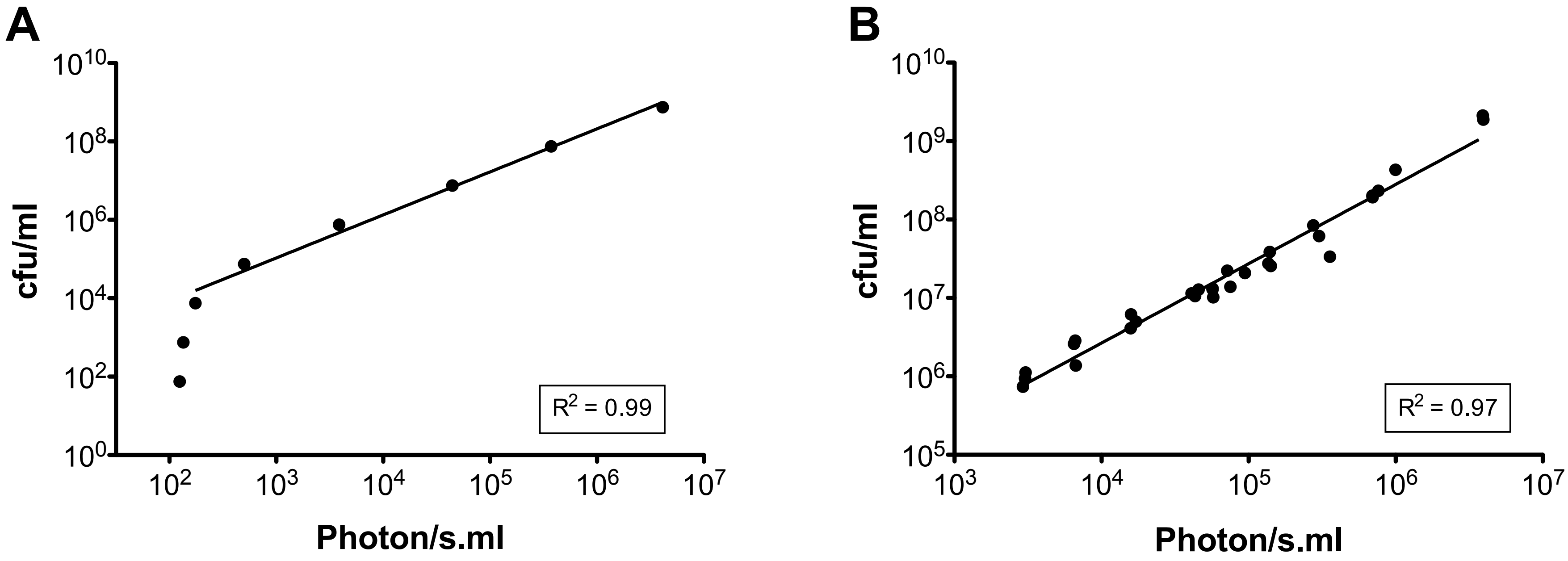

Figure S2. Correlation between bioluminescence emission and cfu counts.
Bacteria were grown at 28°C on agar plates (A) before being suspended in LB and serially diluted, or in broth (B) and aliquots were taken at different time points to determine bacterial counts and light emission. The line represents the least square fit linear regression. The related goodness of fit coefficient (R^2) is indicated on each graph.
