# Supplementary material for: Imaging of Bubonic Plague Dynamics by In Vivo Tracking of Bioluminescent Yersinia pestis
Source: PLoS One. 2012 Apr 5;7(4):e34714. doi: 10.1371/journal.pone.0034714 (PMC3320629; doi:10.1371/journal.pone.0034714)
Supplement: Figure S3 — Temperature-dependent expression of luxCDABE in Y. pestis . Bacteria were grown at 28°C (red circles) or 37°C (blue triangles), in LB broth or on LBH agar plates. Each spot represents the number of photons (count/s) emitted per cfu. The horizontal bar represents the mean light emission per cfu and the vertical bar the standard error. P values were determined with the Mann Whitney test. (PDF) [file pone.0034714.s003.pdf]

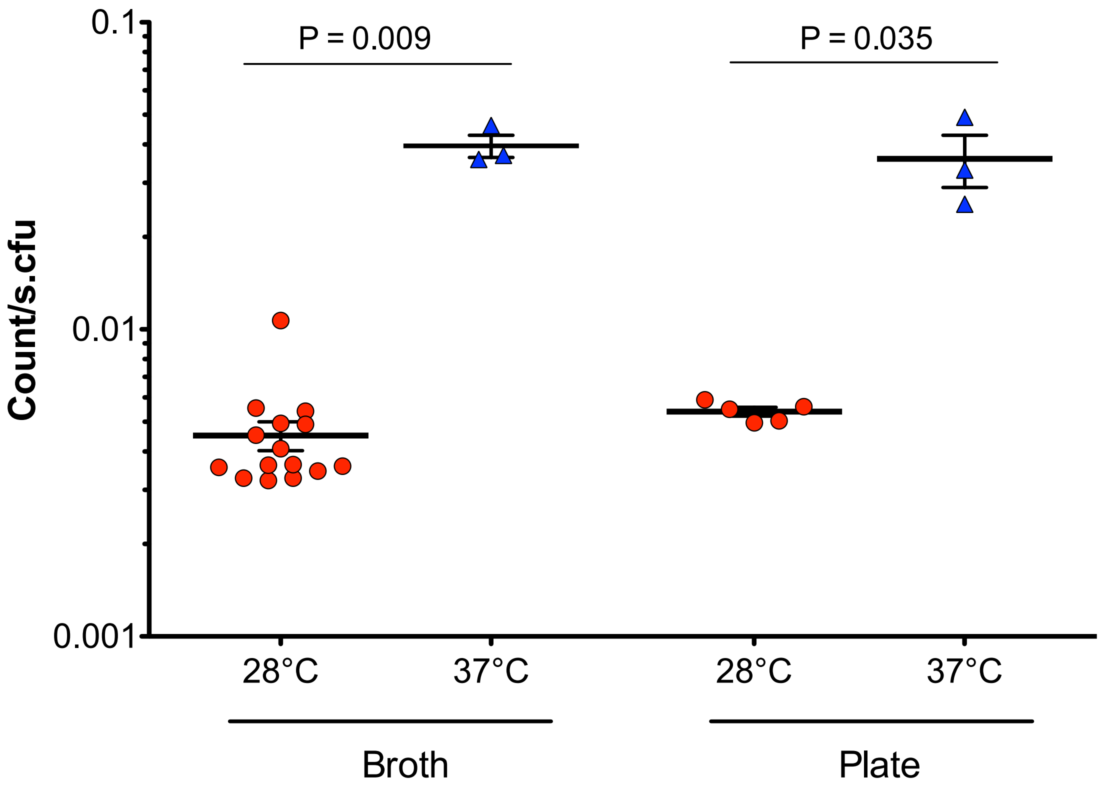

Figure S3. Temperature-dependent expression of *luxCDABE* in *Y. pestis.*
Bacteria were grown at 28°C (red circles) or 37°C (blue triangles), in LB broth or on LBH agar plates. Each spot represents the number of photons (count/s) emitted per cfu. The horizontal bar represents the mean light emission per cfu and the vertical bar the standard error. P values were determined with the Mann Whitney test.
