# Supplementary material for: Imaging of Bubonic Plague Dynamics by In Vivo Tracking of Bioluminescent Yersinia pestis
Source: PLoS One. 2012 Apr 5;7(4):e34714. doi: 10.1371/journal.pone.0034714 (PMC3320629; doi:10.1371/journal.pone.0034714)
Supplement: Figure S4 — Survival curves of mice infected with Y. pestis CO92 or CO92(pLux). Groups of five mice were infected subcutaneously with approximately 10 cfu of Y. pestis CO92 (red line) or bioluminescent Y. pestis CO92(pLux) (blue line) and their mortality was followed daily. (PDF) [file pone.0034714.s004.pdf]

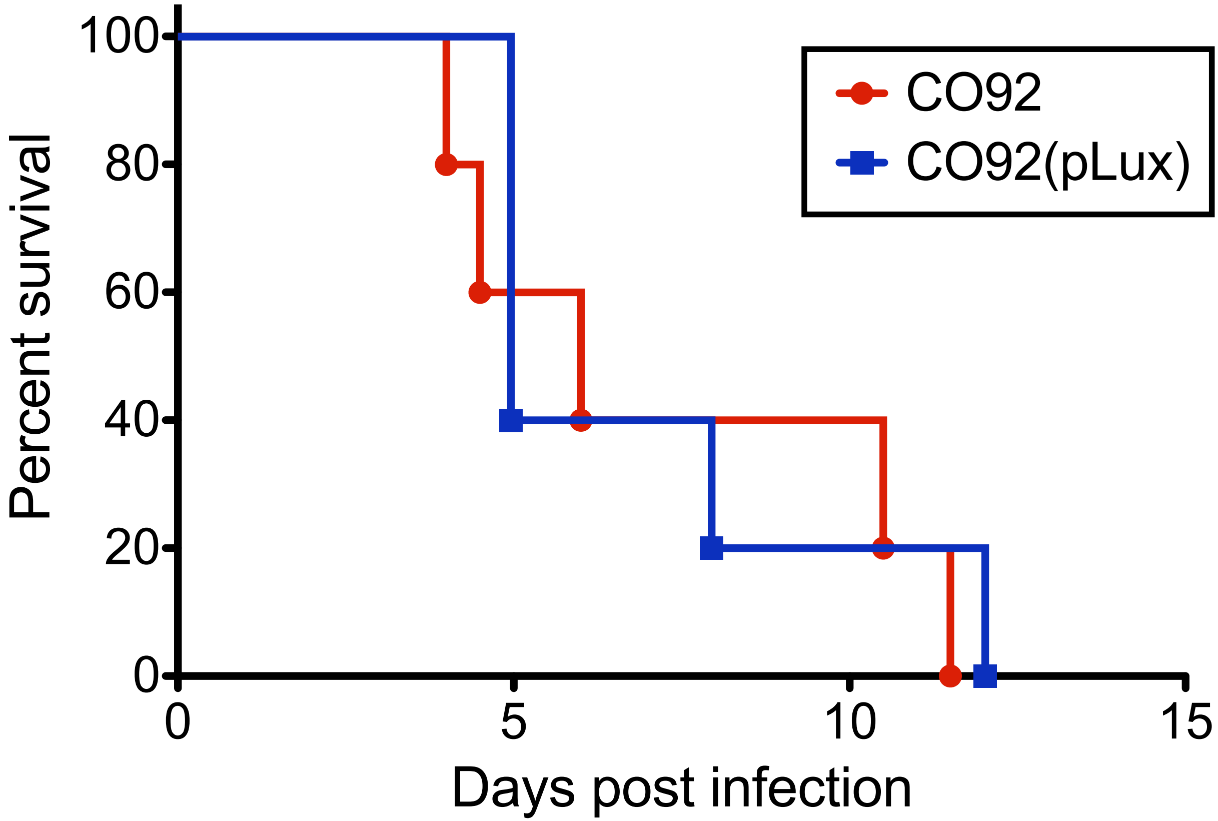


**Figure S4. Survival curves of mice infected with *Y. pestis* CO92 or CO92(pLux)**
Groups of five mice were infected subcutaneously with approximately 10 cfu of *Y. pestis* CO92 (red line) or bioluminescent *Y. pestis* CO92(pLux) (blue line) and their mortality was followed daily.
